# Concentrated ERP for Patients With Difficult‐to‐Treat OCD: Insight as a Predictor of Acute and Long‐Term Outcomes

**DOI:** 10.1155/da/8960147

**Published:** 2025-12-11

**Authors:** Kristian Tjelle, Bjarne Hansen, Stian Solem, Michael G. Wheaton, Gerd Kvale, Kristen Hagen

**Affiliations:** ^1^ Department of Psychiatry, Møre og Romsdal Hospital Trust, Molde, Norway; ^2^ Department of Psychology, Norwegian University of Science and Technology, Trondheim, Norway, ntnu.no; ^3^ Bergen Center for Brain Plasticity, Haukeland University Hospital, Bergen, Norway, helse-bergen.no; ^4^ Department of Psychosocial Sciences, University of Bergen, Bergen, Norway, uib.no; ^5^ Department of Psychology, Barnard College, New York, USA, barnard.edu; ^6^ Department of Clinical Psychology, University of Bergen, Bergen, Norway, uib.no; ^7^ Department of Mental Health, Norwegian University of Science and Technology, Trondheim, Norway, ntnu.no

## Abstract

**Background:**

Cognitive‐behavioral therapy (CBT) with exposure and response prevention (ERP) is a recommended treatment for obsessive‐compulsive disorder (OCD), although many patients who undergo this regimen do not achieve satisfactory symptom relief, and maintaining long‐term remission is challenging. Concentrated exposure therapy (cET) has emerged as a potential treatment for treatment‐resistant OCD. However, the role of insight as a predictor of treatment outcomes in this context remains underexplored.

**Methods:**

We conducted a secondary analysis of a randomized controlled trial (RCT) including 163 adults diagnosed with treatment‐resistant OCD. This study used linear regression models to evaluate whether pre‐treatment levels of insight and changes in insight—measured by the Yale‐Brown Obsessive Compulsive Scale (Y‐BOCS) insight item—were associated with outcomes after 4 days of cET and whether insight levels changed post‐treatment.

**Results:**

Baseline insight did not predict OCD severity at post‐treatment or 3‐month follow‐up but was associated with greater OCD severity at the 12‐month follow‐up. Insight scores improved significantly from pre‐ to post‐treatment but worsened during the follow‐up period. Notably, a change in insight during cET was a significant predictor of OCD symptom severity at all follow‐up intervals.

**Conclusions:**

Baseline insight was not a strong predictor of short‐term treatment outcomes following cET but was related to long‐term OCD severity. Improvements in insight during cET were associated with achieving and maintaining reduced symptoms over time. These results suggest that improvements in OCD and enhanced insight are associated.

**Trial Registration:** ClinicalTrials.gov identifier: NCT02656342.

## 1. Introduction

Individuals diagnosed with obsessive‐compulsive disorder (OCD) typically experience unwanted, intrusive thoughts, images, or urges that are anxiety‐evoking (obsessions). In an effort to cope with these obsessions, they tend to engage in behavioral or mental acts (compulsions) that aim to reduce discomfort and prevent feared consequences [1]. The lifetime prevalence of OCD is estimated to be approximately 1%–3% in the general population [2, 3], with onset typically occurring in early adulthood [4]. OCD is associated with substantial functional impairment, including difficulties in social, occupational, and academic domains, and is linked to a markedly reduced quality of life [5]. Furthermore, instances of recovery without intervention are rare [6, 7].

Cognitive‐behavioral therapy (CBT) involving exposure and response prevention (ERP) is the best documented psychological treatment for OCD. Extensive research supports the efficacy of this treatment at reducing OCD symptoms; however, 40% of patients do not achieve remission from CBT in routine clinical care, and there is a 15% attrition rate [8]. This dropout rate leaves many patients without the full benefit of treatment, and even among those who complete therapy, long‐term follow‐up studies indicate that only approximately half maintain their improvement over time [9, 10], thus highlighting the need for improved therapeutic approaches. Moreover, typical outpatient CBT is typically delivered over the course of 2–3 months [7], but more intensive treatment formats have been developed.

There has been growing interest in the use of concentrated exposure treatment (cET) for treating OCD. The Bergen 4‐day concentrated exposure treatment (B4DT) represents an approach that delivers concentrated ERP‐based treatment over four consecutive days, integrating both group and individual therapy sessions [11]. Several studies have demonstrated high levels of patient acceptability, low dropout rates, and promising clinical outcomes for patients with OCD [11–13], panic disorder [14, 15], and social anxiety disorder [16]. Specifically, studies have reported that approximately 70% of patients are in remission at 4 years after completing treatment [10]. A remission rate of 57% was reported for patients with difficult‐to‐treat OCD (nonresponse and relapse following conventional ERP) [16]. Patients with difficult‐to‐treat OCD constitute a particularly challenging group often referred to as “treatment resistant,” making it especially important to study predictors of treatment response in this population [17–19]. One factor that may significantly influence the success of treatment, particularly in difficult‐to‐treat cases, is the level of insight. Insight refers to patients’ awareness of the irrational nature of their obsessions and their recognition that their compulsive behaviors are excessive or unreasonable [20]. Insight has been associated with earlier OCD onset, longer duration of OCD, more comorbid anxiety and depression [21], and poorer executive functions [22].

The role of insight as a predictor of OCD treatment outcomes has garnered significant attention in the literature. Past research has linked worse insight with greater OCD symptom severity [23, 24], a longer course of the disorder [23–25], and poorer treatment outcomes for both psychological [20, 26–28] and pharmacological interventions [27, 29, 30]. While better insight has generally been associated with more favorable outcomes in ERP, the findings are not consistent [31, 32]. Discrepancies in the literature may be attributed to variations in the assessment of insight, as well as the complexity and potential instability of the construct [33]. Moreover, the influence of insight on treatment outcomes may differ between psychotherapeutic and pharmacological interventions, thus further complicating our understanding of the strength of the relationship between insight and outcome [20, 25–27]. A recent meta‐analysis of the relationship between insight and treatment outcomes in OCD patients revealed a moderate relationship (*r* = 0.33) [34], with even stronger correlations with depressive symptoms (*r* = 0.47).

The potential importance of insight in guiding treatment planning and predicting outcomes remains a significant topic for research, as several important topics remain understudied. In particular, it is necessary to test the links between insight and outcome in different treatment modalities and with different subpopulations of patients. Therefore, this is the first study to investigate the role of insight in cET among a sample of patients with difficult‐to‐treat OCD. We investigated whether pre‐treatment levels of insight predict both short‐ and long‐term outcomes in patients with difficult‐to‐treat OCD who undergo cET. We hypothesized that better baseline insight would be associated with more favorable outcomes, including greater reductions in OCD severity and improved functioning.

Additionally, we sought to investigate whether the degree of insight would change with this intervention. Past research has shown that insight significantly improves along with effective OCD treatment with medications [35, 36]. However, whether insight can change after a brief intensive time interval, such as that exemplified by the 4‐day approach to treatment involved in cET, remains an important unstudied question. Despite its brief duration, this treatment approach has been found to significantly improve OCD symptoms [11, 12]. Therefore, we hypothesized that insight would also improve following cET. Finally, we sought to investigate the relationship between changes in insight and changes in OCD symptom severity over time. We hypothesized that improvements in insight would be associated with improvements in OCD symptom severity during treatment and that changes in insight would also be related to subsequent changes in symptoms as assessed across a 12‐month follow‐up period.

## 2. Methods

### 2.1. Design and Participants

The present study involved secondary data analysis of a previously published randomized controlled trial (RCT) that examined the role of D‐cycloserine (DCS) in treatment outcomes for difficult‐to‐treat OCD patients [37]. The study was triple masked with a placebo‐controlled design and a 12‐month follow‐up. As there were no significant differences between the three conditions (pill placebo and DCS at two different doses), we opted to merge the conditions into one larger sample for this study, thus yielding a total of 163 participants.

The sample (*N* = 163) had a mean age of 35 years, with the majority being female (72%). The participants had been diagnosed with OCD for an average of 16 years. Pre‐treatment symptom severity indicated moderate to severe OCD symptoms. Approximately 45% of the patients were receiving disability benefits, reflecting impaired work ability. Among the participants, 76 were using psychotropic medication, with 32% using SSRIs. Table 1 provides a summary of the pre‐treatment characteristics.

**Table 1:** Pre‐treatment characteristics of the sample.

Characteristics	*M* (SD) (%)
Demographics	
Age	34.60 (10.87)
Female gender	71.8%
OCD onset (year)	18.70 (9.92)
OCD duration (years)	16.17 (10.17)
Single	47.5%
Employment	
Working	34.4%
Student	20.2%
Disability benefits	44.2%
Comorbid disorder	69.3%
Psychotropic medication	46.6%
SSRIs	31.9%
Previous ERP treatment	
Y‐BOCS pre‐treatment	26.83 (5.00)
Y‐BOCS post‐treatment	14.14 (6.05)
Nonresponder	38.7%
Relapse	61.3%

Abbreviations: ERP, exposure and response prevention; OCD, obsessive compulsive disorder; SSRIs, selective serotonin reuptake inhibitors; Y‐BOCS, Yale‐Brown Obsessive Compulsive Scale.

### 2.2. Inclusion Criteria

To be eligible for the study, patients had to meet the DSM‐5 criteria for OCD, be able to undergo treatment as outpatients, be at least 18 years of age, and be fluent in Norwegian. The included patients either responded and later relapsed or did not respond to the treatment for at least six sessions of ERP with a certified therapist to qualify as difficult‐to‐treat patients. A response was defined as a ≥35% reduction and a post‐treatment Yale‐Brown Obsessive Compulsive Score (Y‐BOCS) score of ≤15, followed by a relapse as defined by a ≥35% increase in the Y‐BOCS score from post‐treatment, a Y‐BOCS score of 16 or more, and a CGI‐I score of 6 (“much worse”) or higher. Nonresponders were defined as those with a reduction in Y‐BOCS scores from pre‐ to postintervention of less than 35% and a Y‐BOCS score of ≥16 after treatment. Finally, there had to be a minimum of 4 weeks since the original treatment ended.

### 2.3. Exclusion Criteria

There were a fixed set of exclusion criteria for participating in the study, including patients with ongoing substance abuse/dependance, bipolar disorder or psychosis, suicidal ideation or plans, intellectual disability (based on previous medical history), and living >1 h drive by car/train from the treatment location. Participants who were unwilling to refrain from anxiety‐reducing substances, such as anxiolytics (e.g., benzodiazepines) and alcohol, during the 2 days of ERP were also excluded. Additionally, patients were also excluded if their antidepressants had not been stabilized for at least 12 weeks or if they were unwilling to remain on stable dosages during the four intervention days. Additional exclusion criteria related to the study were pregnancy or breast feeding, hypersensitivity to DCS, renal impairment, and porphyria.

### 2.4. Determination of Eligibility

All patients were screened for inclusion via the Y‐BOCS and Mini International Neuropsychiatric Interview (MINI), and the inclusion/exclusion criteria are listed. Patients receiving a preliminary OCD diagnosis after the MINI had a diagnostic interview using the SCID‐5 by a national team of independent assessors. Questionable cases were evaluated individually by three senior investigators who, after reaching a consensus, made the final decision of inclusion or exclusion from the study.

### 2.5. Adherence and Competence

All therapists were trained through the national OCD training program, led by internationally recognized experts, and had prior experience with ERP in specialized OCD clinics. Each treatment group was led by a senior clinician who had delivered a minimum of five previous B4DT groups. The intervention followed a standardized manual. The psychoeducation module was delivered using a fixed PowerPoint presentation with predefined slide content. Daily therapist team meetings were conducted to coordinate exposure tasks and maintain treatment fidelity. All sessions and therapist meetings were video recorded. Each group included a trained therapist observer who monitored adherence to the protocol, and no deviations were reported. Recordings were subsequently reviewed and rated by two experts in B4DT who had not been involved in the delivery of the respective groups. Using a standardized 3‐point scale (0 = not adherent/incompetent, 1 = partially adherent/competent, and 2 = fully adherent/competent), all groups were rated as adherent and competently delivered (score = 2), with the exception of one group, which was rated as partially adherent and competent (score = 1).

### 2.6. Primary Outcome Measure

The Y‐BOCS [38, 39] is considered the gold standard for assessing OCD symptoms [40]. The scale involves an interview with a certified clinician and encompasses a total of 19 separate parameters, each rated on a 5‐point scale that spans from 0 (indicating the absence of symptoms) to 4 (indicating the presence of severe symptoms). The first 10 parameters give an overall OCD symptom severity score that falls within the range of 0–40. Furthermore, it breaks down into subscores for obsessions, with a range of 0–20, and compulsions, which range from 0 to 20 [38, 39].

Insight was measured using item 11 of the Y‐BOCS, which addresses both obsessive thoughts and compulsive behaviors. Items 11–19 of the Y‐BOCS are investigative questions and are not included in the total Y‐BOCS score, but they can provide valuable information in the assessment of symptoms. For the insight item, the interviewer asks patients if they think the concerns or behaviors are reasonable, what they think would happen if they did not perform the compulsion, and if they are convinced that something would truly happen. Patient insight into the senselessness or excessiveness of the OCD is measured on a 0–4 scale: 0 = excellent insight, fully rational; 1 = good insight, readily acknowledging the absurdity or excessiveness of thoughts or behaviors but not completely convinced that there is not something besides anxiety to be concerned about; 2 = fair insight, reluctantly admitting thoughts or behavior seem unreasonable or excessive but wavers (may have unrealistic fears but no fixed convictions); 3 = poor insight, maintaining that thoughts or behaviors are not unreasonable or excessive but acknowledge the validity of contrary evidence; and 4 = delusional insight, convinced that concerns and behavior are reasonable and unresponsive to contrary evidence.

### 2.7. Secondary Outcome Measures

The Work and Social Adjustment Scale ([WSAS] [41]) is a questionnaire comprising five items. These items assess an individual’s level of impairment in various life domains, including work, social interactions, private activities, home functioning, and close relationships. The respondents evaluate each item independently on a 9‐point scale ranging from 0 (indicating no impairment) to 8 (signifying very severe impairment). Assessments were conducted prior to treatment and at the 1‐year follow‐up. Consequently, total WSAS scores range from 0 to 40, with higher scores indicating greater degrees of impairment in functioning. Importantly, the WSAS has exhibited strong internal consistency and test–retest reliability [42].

### 2.8. Statistical Analysis

Spearman’s rho correlation coefficients were utilized to test the zero‐order associations between pre‐treatment insight, OCD symptom severity (Y‐BOCS), and functional impairment (WSAS) across time points. Pearson’s correlation coefficients were calculated to assess these relationships, with significance levels set at *p* < 0.05.

A series of linear regression analyses were performed to examine whether pre‐treatment insight predicted treatment outcomes, as measured by Y‐BOCS scores at post‐treatment, 3‐month follow‐up, and 12‐month follow‐up. The models included pre‐treatment Y‐BOCS scores and insight scores as predictors, and the statistical significance of the models and individual predictors are reported.

To assess changes in insight over time, a repeated measures general linear model (GLM) with Greenhouse–Geisser correction was employed. This analysis examined whether insight scores differed significantly across the four time points, and post hoc Bonferroni‐corrected comparisons were conducted to identify specific time points where changes occurred.

Finally, hierarchical regression analyses were run to determine whether changes in insight from pre‐ to post‐treatment were associated with post‐treatment and follow‐up Y‐BOCS scores. These analyses included residualized change scores in insight and Y‐BOCS scores as predictors, with statistical significance levels reported for the overall models and individual predictors.

Missing data in this dataset were handled using the expectation maximization (EM) method in SPSS version 29, and the proportion of missing data was 9.8%. The EM method is adequate for datasets with less than 25% missing data that are missing completely by random, criteria that were met in this study (Little’s MCAR test; *x* ^2^ = 70,167, DF = 102, *p* = 0.993).

## 3. Results

### 3.1. Descriptive Statistics

The sample consisted of 163 patients. OCD‐related insight was reported as excellent for 58.3% (*n* = 95), good for 26.4% (*n* = 43), fair for 12.9% (*n* = 21), poor for 2.5% (*n* = 4), and delusional for 0% of the participants. The Y‐BOCS total scores and Y‐BOCS insight scores at the four assessments are presented in Table 2.

**Table 2:** Descriptive statistics for Y‐BOCS and insight (Y‐BOCS Q11).

Variable	*M*	SD	*d*_pre-*x*_
Y‐BOCS pre‐treatment	27.03	3.86	—
Y‐BOCS post‐treatment	12.39	5.89	2.94
Y‐BOCS 3‐month follow‐up	13.83	7.18	2.29
Y‐BOCS 12‐month follow‐up	14.36	7.15	2.21
Insight pre‐treatment	0.59	0.81	—
Insight post‐treatment	0.33	0.59	0.37
Insight 3‐month follow‐up	0.48	0.72	0.14
Insight 12‐month follow‐up	0.53	0.74	0.08

*Note:* Insight is measured using Y‐BOCS item 11. Cohen’s *d* values were calculated using pooled standard deviations.

Abbreviation: Y‐BOCS, Yale‐Brown Obsessive Compulsive Scale.

The correlations between pre‐treatment insight and OCD symptom severity and functional impairment over time are summarized in Table 3. As shown, insight assessed prior to treatment were not significantly correlated with OCD severity at pre‐treatment, post‐treatment, or 3‐month follow‐up, suggesting that initial levels of insight were not related to OCD severity at these timepoints. However, insight was significantly correlated with OCD severity at 12 months (*r* = 0.21, *p* < 0.01), with worse insight associated with greater OCD severity 1 year later. Insight was not correlated with functional impairment (WSAS) at pre‐treatment or at the 12‐month follow‐up.

**Table 3:** Correlations between insight, OCD symptom severity, and functional impairment.

Measure	1	2	3	4	5	6	7	8	9	10
Insight										
1. Pre	—	0.425^∗∗^	0.353^∗∗^	0.287^∗∗^	0.045	−0.016	0.020	0.194^∗^	0.072	0.094
2. Post	—	—	0.615^∗∗^	0.449^∗∗^	0.054	0.253^∗∗^	0.339^∗∗^	0.347^∗∗^	0.123	0.308^∗∗^
3. 3‐month	—	—	—	0.434^∗∗^	0.123	0.296^∗∗^	0.372^∗∗^	0.432^∗∗^	0.115	0.276^∗∗^
4. 12‐month	—	—	—	—	−0.126	0.275^∗∗^	0.255^∗∗^	0.362^∗∗^	−0.101	0.248^∗∗^
Y‐BOCS										
5. Pre	—	—	—	—	—	0.149	0.268^∗∗^	0.154^∗^	0.310^∗∗^	0.131
6. Post	—	—	—	—	—	—	0.636^∗∗^	0.357^∗∗^	0.167^∗^	0.368^∗∗^
7. 3‐month	—	—	—	—	—	—	—	0.617^∗∗^	0.202^∗∗^	0.639^∗∗^
8. 12‐month	—	—	—	—	—	—	—	—	0.188^∗^	0.817^∗∗^
WSAS										
9. Pre	—	—	—	—	—	—	—	—	—	0.486^∗∗^
10. 12‐month	—	—	—	—	—	—	—	—	—	—

*Note:* Insight, insight item on Y‐BOCS (Q11).

Abbreviations: WSAS, Work and Social Adjustment Scale; Y‐BOCS, Yale‐Brown Obsessive‐Compulsive Scale.

^∗∗^ *p* < 0.01.

^∗^ *p* < 0.05.

Insight at pre‐treatment was significantly correlated with insight at post‐treatment (*r* = 0.41, *p* < 0.01), 3‐month follow‐up (*r* = 0.36, *p* < 0.01), and 12‐month follow‐up (*r* = 0.32, *p* < 0.01). Post‐treatment insight was correlated with insight at the 3‐month (*r* = 0.67, *p* < 0.01) and 12‐month follow‐up (*r* = 0.51, *p* < 0.01). Insight at the 3‐month follow‐up was also correlated with insight at the 12‐month follow‐up (*r* = 0.55, *p* < 0.01) (Table 2).

### 3.2. Does Pre‐Treatment Insight Predict Treatment Outcome?

A series of linear regression analyses were conducted to predict post‐treatment and follow‐up Y‐BOCS scores using pre‐treatment Y‐BOCS scores and insight as predictors. The model predicting post‐treatment Y‐BOCS scores was not significant (*R* ^2^ = 0.02, *F* (2, 160) = 1.90, *p* = 0.153), with neither Y‐BOCS (*β* = 0.15, *p* = 0.053) nor insight (*β* = 0.00, *p* = 0.969) emerging as significant predictors. At the 3‐month follow‐up, the model showed a better fit (*R* ^2^ = 0.07, *F* (2, 160) = 6.16, *p* = 0.003), with Y‐BOCS scores being a significant predictor (*β* = 0.26, *p* < 0.001), although insight remained nonsignificant (*β* = 0.03, *p* = 0.662). At the 1‐year follow‐up, the model was significant and explained a modest amount of variance (*R* ^2^ = 0.06, *F* (2, 160) = 5.49, *p* = 0.005), with insight becoming a significant predictor (*β* = 0.20, *p* = 0.010) and Y‐BOCS scores being marginally significant (*β* = 0.15, *p* = 0.056).

### 3.3. Does Insight Improve after cET?

**Figure 1:**
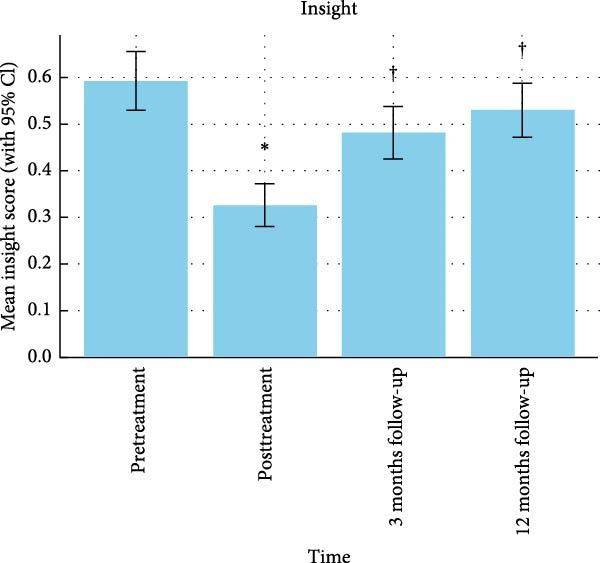
Y‐BOCS insight means and standard errors at all four time points.  ^∗^ = significant difference from pre‐treatment and † = significant difference from post‐treatment.

Insight scores were compared across the four time points using a repeated measures GLM with Greenhouse–Geisser correction. There was a significant overall effect of time, *F* (2.5, 404.95) = 7.47, *p* < 0.001, partial eta squared = 0.164. Post hoc Bonferroni‐corrected comparisons of the individual time points revealed a significant improvement in insight from pre‐treatment to post‐treatment (*p* < 0.001), followed by a significant decline in insight from post‐treatment to the 3‐month follow‐up (*p* = 0.002). There was no significant change in insight scores from the 3‐month to the 12‐month follow‐up (see Figure 1).

### 3.4. Is a Change in Insight Associated with Treatment Outcome?

The regression predicting post‐treatment Y‐BOCS scores, which entered pre‐treatment Y‐BOCS scores and residualized change scores in insight from pre‐ to post‐treatment as predictors, was significant, *R* ^2^ = 0.09, *F* (2, 160) = 7.97, *p* < 0.001. The residual score for insight emerged as a significant predictor, whereas the baseline Y‐BOCS score did not. The beta weight associated with the change in insight indicated that greater improvement in insight was associated with lower post‐treatment Y‐BOCS symptoms (*β* = 0.26, *p* < 0.001), thus highlighting an association between changes in insight and symptom changes during treatment.

A second multiple regression analysis was conducted to predict Y‐BOCS scores at the 3‐month follow‐up, using pre‐treatment Y‐BOCS scores and residualized change scores in both insight and Y‐BOCS scores from pre‐ to post‐treatment as predictors. The overall model was significant, *R* ^2^ = 0.46, *F* (3, 159) = 45.35, *p* < 0.001, explaining 46.1% of the variance in Y‐BOCS scores at the 3‐month follow‐up. Within the model, both the residualized change in Y‐BOCS scores from pre‐ to post‐treatment (*β* = 0.56, *p* < 0.001) and pre‐treatment Y‐BOCS scores (*β* = 0.26, *p* < 0.001) emerged as significant predictors, indicating that higher pre‐treatment severity and greater reduction in Y‐BOCS scores were associated with lower severity at the 3‐month follow‐up. Additionally, the residualized change in insight also emerged as a significant predictor (*β* = 0.17, *p* = 0.005), suggesting that improvement in insight from pre‐ to post‐treatment was associated with lower Y‐BOCS scores at the 3‐month follow‐up.

A third multiple regression analysis was conducted to predict Y‐BOCS scores at the 12‐month follow‐up; baseline Y‐BOCS scores and residualized change scores in both insight and Y‐BOCS scores from pre‐ to post‐treatment were used as predictors. The overall model was significant, *R* ^2^ = 0.17, *F* (3, 159) = 11.07, *p* < 0.001, explaining 17.3% of the variance in Y‐BOCS scores at the 12‐month follow‐up. Within the model, the residualized change in Y‐BOCS scores from pre‐ to post‐treatment (*β* = 0.30, *p* < 0.001) emerged as a significant predictor, indicating that greater reductions in Y‐BOCS scores were associated with lower severity at the 12‐month follow‐up. Pre‐treatment Y‐BOCS scores also contributed significantly to the model (*β* = 0.15, *p* = 0.036), suggesting that initial severity still had a lingering impact on long‐term outcomes. Additionally, the residualized change in insight from pre‐ to post‐treatment was a significant predictor (*β* = 0.18, *p* = 0.020), highlighting that improvements in insight were associated with better long‐term outcomes in terms of OCD severity. Table 4 summarizes the results of the regression analyses.

**Table 4:** Summary of hierarchical regression analyses predicting OCD symptoms.

Independent variables	*β*	*B*	SE *B*	*p*
Y‐BOCS post‐treatment				
Y‐BOCS pre‐treatment	0.15	0.22	0.12	0.056
Δ Insight	0.26	2.83	0.82	<0.001
Y‐BOCS 3‐month follow‐up				
Y‐BOCS pre‐treatment	0.26	0.49	0.11	<0.001
Δ Insight	0.17	2.27	0.80	0.005
Δ Y‐BOCS	0.56	0.69	0.07	<0.001
Y‐BOCS 12‐month follow‐up				
Y‐BOCS pre‐treatment	0.15	0.028	0.13	0.036
Δ Insight	0.18	2.33	0.99	0.020
Δ Y‐BOCS	0.30	0.37	0.09	<0.001

Abbreviation: Y‐BOCS, Yale‐Brown Obsessive‐Compulsive Scale.

## 4. Discussion

This study explored the relationship between pre‐treatment insight and the severity of OCD over time, as well as the predictive value of changes in insight following cET for treatment outcomes. The findings highlight several important aspects of the role that insight plays in the treatment and long‐term management of OCD with this brief intervention.

At pre‐treatment, the majority of participants exhibited excellent insight, with only a small percentage displaying poor insight and no patients with exhibiting absent/delusional insight. Importantly, lower levels of insight pre‐treatment were associated with greater OCD severity 1 year later. However, pre‐treatment insight did not show a significant relationship with OCD severity during the earlier stages of treatment, including at pre‐treatment, post‐treatment, and 3‐month follow‐up. This finding suggests that while initial levels of insight may not be immediately related to treatment outcomes, insight could have a longer‐term effect on the course of the disorder. This finding corresponds with previous research [17, 21–23] and a meta‐analysis suggesting an association between insight and outcome in ordinary OCD treatment [28]. Additionally, insight did not show significant correlations with functional impairment, highlighting that insight may be more relevant to OCD severity rather than overall functioning. However, a meta‐analysis revealed stronger correlations between insight and depression than with OCD [28], which suggests that insight could have different effects depending upon the choice of outcome measure. This finding could explain some of the discrepancy in previous research [26, 27] along with the complexity in assessing insight [30].

From a clinical perspective, the results from this study indicate that cET is an appropriate treatment choice for patients with both excellent and poor insight, as worse insight does not predict worse acute outcomes. However, the finding that baseline insight was significantly correlated with OCD severity at 12 months may indicate that patients with worse pre‐treatment insight might be at risk for worsening of symptoms (i.e., relapse) during long‐term follow‐up. This could be related to insight being associated with depression [28] and executive functions [22] and should be a topic for further investigations.

Our results also demonstrated that insight significantly improved in the acute time frame following cET. The effects were small–moderate from pre‐ to post‐treatment. However, the results also revealed a significant worsening of insight during the follow‐up period, indicating a potential decline in the gains made during treatment. This fluctuation in insight over time underscores the dynamic nature of insight in OCD and suggests that sustained interventions might be necessary to maintain improvements in insight. Future work should investigate whether deterioration in insight may precede and predict subsequent worsening of global OCD symptoms. Importantly, relapse prevention efforts might be designed to carefully monitor for changes in insight, as patients who become less insightful about the sensitivity of their OCD symptoms (i.e., that compulsions are excessive and not realistically linked to preventing feared consequences) may be at greater risk for experiencing a relapse.

Our results also revealed that changes in insight during the brief period of treatment (i.e., 4 days of cET) were a significant predictor of OCD symptoms across time. Although the pre‐treatment degree of insight did not strongly predict post‐treatment OCD severity, improvements in insight during treatment were associated with better treatment outcomes, not only immediately after treatment, but also at long‐term follow‐up (both 3 and 12 months). This finding indicates the relevance of addressing and improving insight during treatment as a potential therapeutic mechanism of ERP, as changes in insight are significantly related to treatment outcome over time. These findings underscore that while initial OCD severity plays a role, the gains made during treatment in both symptom severity and insight could be critical for long‐term management. However, changes in insight could also be a byproduct of improvement in OCD symptoms.

The present results contribute new knowledge to the literature on how OCD‐related insight relates to treatment with cET, which has not previously been studied. However, several limitations of the present study should also be mentioned. First, this study was a secondary data analysis of a previously published trial and was not specifically designed to test insight as a predictor. As such, the assessment of insight was limited to a single item on the Y‐BOCS, whereas other studies have employed more thorough assessments of insight, such as the Brown Assessment of Beliefs Scale ([BABS] [43]). The BABS assesses insight across multiple dimensions, including conviction, others’ views, explanations of differing views, fixity of beliefs, attempts to disprove beliefs, and beliefs in psychiatric causes. The overvalued ideas scale (OVIS) is another measure of insight [44] that assesses the strength of belief, reasonableness, lowest and highest strength of belief, accuracy, adherence by others, views of others, effectiveness of compulsion, insight, strength of resistance, and duration of belief. Therefore, the BABS and OVIS could provide more nuanced profile of insight and may yield stronger predictive validity [33]. However, the OVIS and BABS have shown strong correlations with the insight item of the Y‐BOCS [33, 45], but the BABS has stronger predictive validity than the Y‐BOCS [45]. Given the conceptual and methodological limitations of assessing insight with a single item, interpretations of its predictive role in this study should be made with caution. Future research should incorporate multidimensional instruments, such as the BABS and OVIS, to more accurately capture the complexity of insight in OCD.

Additionally, our sample was somewhat limited in that the participants tended to have excellent or good insight. Relatively few patients had poor insight, and no participants were rated as having absent/delusional insight. Absent/delusional insight appears to be rare in OCD; however, future research is needed to determine whether cET will also be effective for patients with this degree of insight, as the present results may not be generalizable to these patients. Finally, the present sample was composed of Norwegian adults; therefore, future work is needed to replicate these results in more diverse samples of patients, including both international and pediatric samples.

## Ethics Statement

The study was approved by the Regional Committee for Medical and Health Research Ethics (REK: 2013/195). Written informed consent was obtained from all participants. All procedures were carried out in accordance with relevant guidelines and regulations, including the Declaration of Helsinki.

## Consent

The authors have nothing to report.

## Disclosure

All authors (Kristian Tjelle, Bjarne Hansen, Stian Solem, Michael G. Wheaton, Gerd Kvale, and Kristen Hagen) have read and approved the final version. After using the ChatGPT tool, the authors reviewed and edited the content as needed and take full responsibility for the final manuscript. The funders had no role in the design, execution, analysis, or reporting of the study.

## Conflicts of Interest

The authors declare no conflicts of interest.

## Author Contributions

Kristian Tjelle, Bjarne Hansen, Stian Solem, Michael G. Wheaton and Kristen Hagen contributed to the conceptualization and design of the study. Kristian Tjelle, Stian Solem and Kristen Hagen prepared the data. Kristian Tjelle, Stian Solem, and Kristen Hagen conducted the formal analyses. Kristian Tjelle drafted the original manuscript. All authors (Kristian Tjelle, Bjarne Hansen, Stian Solem, Michael G. Wheaton, Gerd Kvale, and Kristen Hagen) contributed to the writing and editing of the manuscript. Kristen Hagen served as the main supervisor of the project.

## Funding

The original project was funded by the Research Council of Norway (project number: 243675, HELSEFORSK). The Bergen Center for Brain Plasticity is funded by the Trond Mohn Foundation, the Kavli Trust, Haukeland University Hospital, and the University of Bergen. The research was conducted independently of the funders to ensure scientific integrity. Kristian Tjelle was funded from Samarbeidsorganet Helse Midt‐Norge (Grant 2018/42794‐25).

## Data Availability

The data that support the findings of this study are not publicly available due to privacy and ethical restrictions. However, deidentified data may be made available upon reasonable request to the corresponding author, subject to approval by the data controller at Haukeland University Hospital. Data are stored in a secure, controlled‐access repository in accordance with institutional and ethical guidelines.
